# Visualization of early prostatic adenocarcinoma as a stem cell disease

**DOI:** 10.18632/oncotarget.12709

**Published:** 2016-10-18

**Authors:** Maggie Y. Jiang, Tammy L. Lee, Su-Shin Hao, Sepi Mahooti, Stephen M. Baird, Daniel J. Donoghue, Martin Haas

**Affiliations:** ^1^ Moores UCSD Cancer Center, University of California San Diego, La Jolla, CA 92093, USA; ^2^ Department of Pathology, University of California San Diego, La Jolla, CA 92093, USA; ^3^ Division of Biological Sciences, University of California San Diego, La Jolla, CA 92093, USA; ^4^ Department of Chemistry and Biochemistry, University of California San Diego, La Jolla, CA 92093, USA

**Keywords:** cancer stem cell, prostatic adenocarcinoma, prostatic intraepithelial neoplasia, stem cell antigen

## Abstract

Prostate Cancer represents the second leading cause of cancer death among men in the United States, and the third leading cause of cancer death among men in Europe. We have previously shown that cells possessing Cancer Stem Cell (CSC) characteristics can be grown from human PrCa tissue harvested at the time of prostatectomy. However, the cellular origin of these CSCs was not previously known. In most cases, simple hematoxylin and eosin (H&E) stained sections are sufficient to make a definitive diagnosis of prostatic adenocarcinoma (PrCa) in needle biopsy samples. We utilized six different antibodies specific for stem cell antigens to examine paraffin sections of PrCa taken at the time of needle-biopsy diagnosis. These antisera were specific for CD44, CD133, ALDH7A1, LGR-5, Oct-4 and NANOG. We demonstrate specific staining of tumor cells with all six antisera specific for stem cell antigens. Some of these antibodies also react with cells of hyperplastic glands, but the patterns of reactivity differ from those of malignant glands. These findings demonstrate that at the time of diagnosis, PrCa consists of cells exhibiting properties of CSCs and consistent with the possibility that PrCa is a stem cell disease.

## INTRODUCTION

The notion that Cancer Stem Cells (CSCs) are the cells of origin of prostate cancer has been widely reported [1, 2]. Some work has suggested that luminal glandular cells of the prostate rather than basal stem cells are the source of prostate cancer initiating cells [3] although this remains unclear. Whether prostate cancer stem cells (PrCSCs) play a role in the initiation of prostate cancer or in its progression to Castration Resistant Prostate Cancer (CRPC), or both, also remains ambiguous. Current models suggest that prostate cancers are initiated by the development of prostatic intraepithelial neoplasia (PIN), becoming locally invasive adenocarcinoma, followed by metastatic androgen dependent- and, finally, metastatic CRPC [4–6]. The stepwise model of prostate cancer, the result of many years of histopathologic studies, now hypothesizes that early phases harbor small numbers of androgen-unresponsive CSCs which are not affected by therapies that target androgen, its receptor or its pathways [7]. Clearly, the differentiated descendants of CSCs, which at the time of prostatectomy comprise the bulk of the cancer, are responsive to androgen-deprivation therapies. However, while the bulk of the tumor mass shrinks after androgen-deprivation treatments, CSCs in the early cancerous prostate may survive and re-appear as androgen pathway-unresponsive cells during progression. Small numbers of these cancer-initiating stem cells could therefore drive the early cancer and become an increasing source of treatment-resistant prostate cancer during progression. Treatment-failure from androgen-deprivation would thus hinge on the persistence of unresponsive CSCs [7].

We have previously shown that cells possessing CSC characteristics can be grown from human PrCa tissue harvested at the time of prostatectomy in patients clinically staged as I or II, stages at which the cancer cells remain resident in the prostate [7]. Characteristics of these PrCSCs, cultured from tissue obtained during prostatectomy, include: (1) propagation in the absence of androgen; (2) expression of multiple markers thought to be characteristic of stem cells [7]; (3) growth in suspension as “spheres” [8]; and (4) ability to generate (human) prostate cancers following orthotopic xenografting into the anterior prostates of SCID mice [7].

The cellular origin and the time of appearance of these PrCSCs remain enigmatic. Stem cell signaling pathways and stem cell characteristics may be acquired during the early carcinogenic process [9]. If mutated luminal, neuroendocrine or other differentiated cells are a source of CSCs in the early cancerous prostate, the derivation of a cancer stem cell-like phenotype could involve a mechanism of “de-differentiation” and/or “re-programming” [10] to pluripotency as part of the carcinogenic process. How early in the sequence of events of PrCa development are PrCSCs identifiable and what is their source?

Isolation of PrCSCs from prostatectomy-derived cancer tissue clearly demonstrates their presence in low stage disease, especially since these cells generate human prostate cancers in SCID mice [7]. However, the source of these tumorigenic CSCs is not known. Prostatectomy-derived CSCs may be re-programmed to multipotency or even pluripotency by a cancer-associated re-programming mechanism. Alternatively, these CSCs may be derived from pre-existing normal prostate basal stem cells, presumably by mutation.

## RESULTS

We sought to identify cells that could be classified as cancer stem-like cells in the low-stage cancerous prostate by staining needle biopsy samples with six widely recognized antibodies to stem cell markers. The expression in needle biopsy tissue of multiple stem cell markers might be interpreted as evidence for cellular stemness, especially if multiple recognized stem cell markers are present in the same H&E-stained pathological structures routinely used by anatomical pathologists as diagnostic evidence for PrCa. We found that serial sections of these same cancer areas reacted with all six of the stem cell-specific antibodies studied. While the expression of a single stem cell marker may not specify the stemness of the reactive cells, the use of six widely-recognized stem cell-specific markers, each staining carcinomatous glands, demonstrates the stem-like characteristics shared by the cells in the malignant glands. Antibodies specific for the markers CD44 [11], CD133 [12], ALDH7A1 [13], LGR-5 [14], Oct-4 [15] and NANOG [16] were used. Though not all six markers were tested on all eight needle-biopsy cancer samples due to the limited number of sections that could be cut from the needle biopsy material available after histopathological diagnosis, all prostate cancer tissues specifically reacted with at least five of the six stem cell marker antibodies. Thus, at the level of histology of needle biopsy tissue, the stem cell-like properties of the cells forming the carcinomatous glands are evident. Significantly, normal prostate tissue in-between the two to eight areas of adenocarcinoma or hyperplasia within the biopsy tissue was not reactive with the antibodies tested.

Table 1 summarizes the pathology of the needle biopsy tissues obtained for the current experiments. We studied eight paraffin blocks of anonymous prostate needle biopsies previously used for clinical diagnoses and no longer needed clinically. Blocks were cut into 5 μm thick sections, yielding 5-8 usable serial sections each. Seven paraffin blocks yielded a diagnosis of PrCa, while one yielded a diagnosis of benign prostatic hyperplasia (BPH).

**Table 1:** Needle biopsy samples. Eight cases were processed histologically (H&E staining) and the presence of cancer was established in 7 of 8 samples as shown below. All samples with adenocarcinoma also contained examples of benign hyperplasia

Patient	Diagnosis (Biopsy)	Gleason Score
A1	Adenocarcinoma + hyperplasia	3 + 3
C1	Adenocarcinoma + hyperplasia	3 + 3
E1	Adenocarcinoma + hyperplasia	3 + 3
F1	Adenocarcinoma + hyperplasia	3 + 3
I1	Hyperplasia	*
J1	Adenocarcinoma + hyperplasia	3 + 3
K1	Adenocarcinoma + hyperplasia	3 + 3
L1	Adenocarcinoma + hyperplasia	3 + 3

In each of these cases, multiple areas with cancer (minimum of 2, maximum of 5) were stained with fluorescent stem cell-specific antibodies then observed and photographed by fluorescent microscopy. Patient sample I-1 did not contain adenocarcinoma but only hyperplasia.

**Figure 1:**
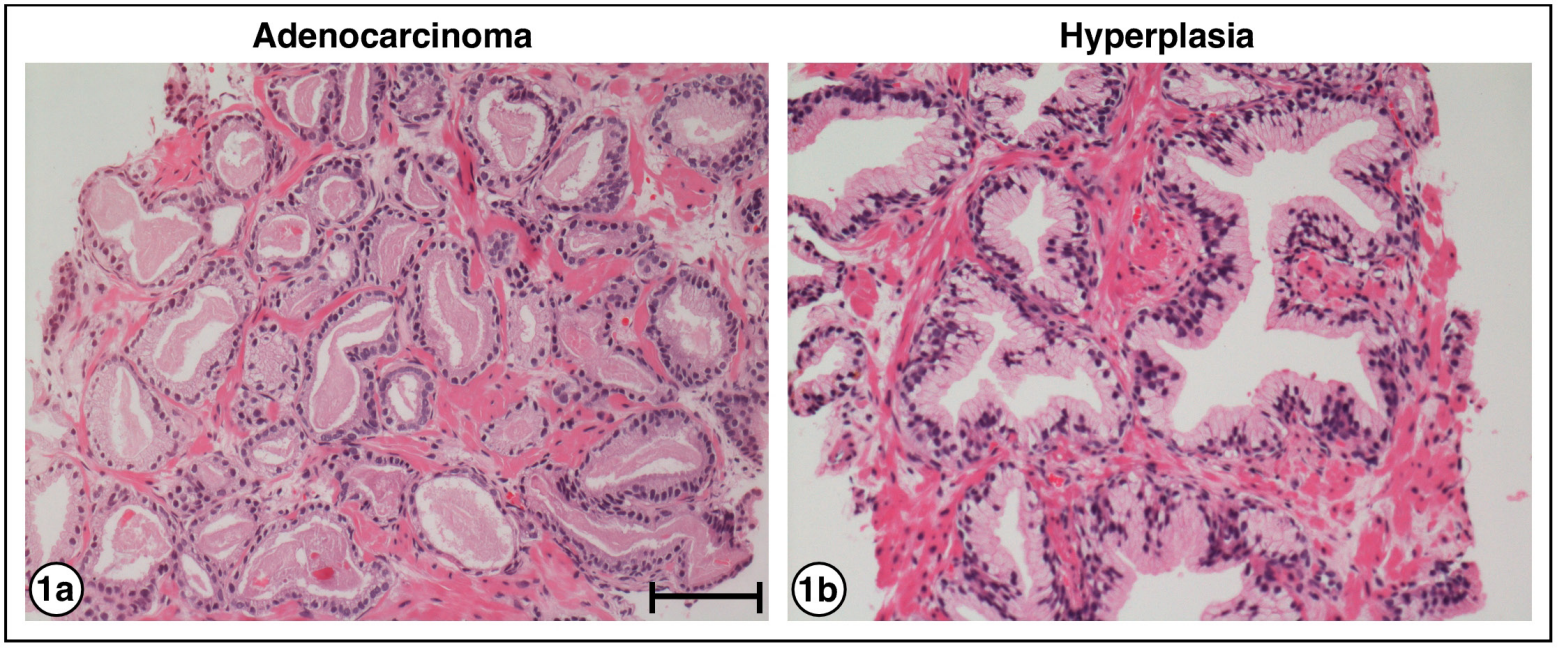
H&E staining of PrCa and BPH **a.** Adenocarcinoma of the prostate, patient A1, Gleason grade 6, showing small glands, single layers of nuclei, and lack of polarity of nuclei within the cells. **b.** Hyperplasia, patient I1, showing glands with complex infolding, double layers of nuclei, and basal polarization of nuclei within cells. Bar = 50 μm.

Figure 1a is an example of an H&E stained needle biopsy tissue of PrCa from patient A1, Gleason grade 6. It shows small glands, single layers of nuclei, a dearth of myoepithelial cells and a lack of polarity of nucleated cells within the glands. Figure 1b shows an H&E-stain of a benign hyperplasia consistent with BPH (patient I1). The glands demonstrate complex infolding, double layers of nuclei, and basal polarization of nuclei within cells.

**Figure 2:**
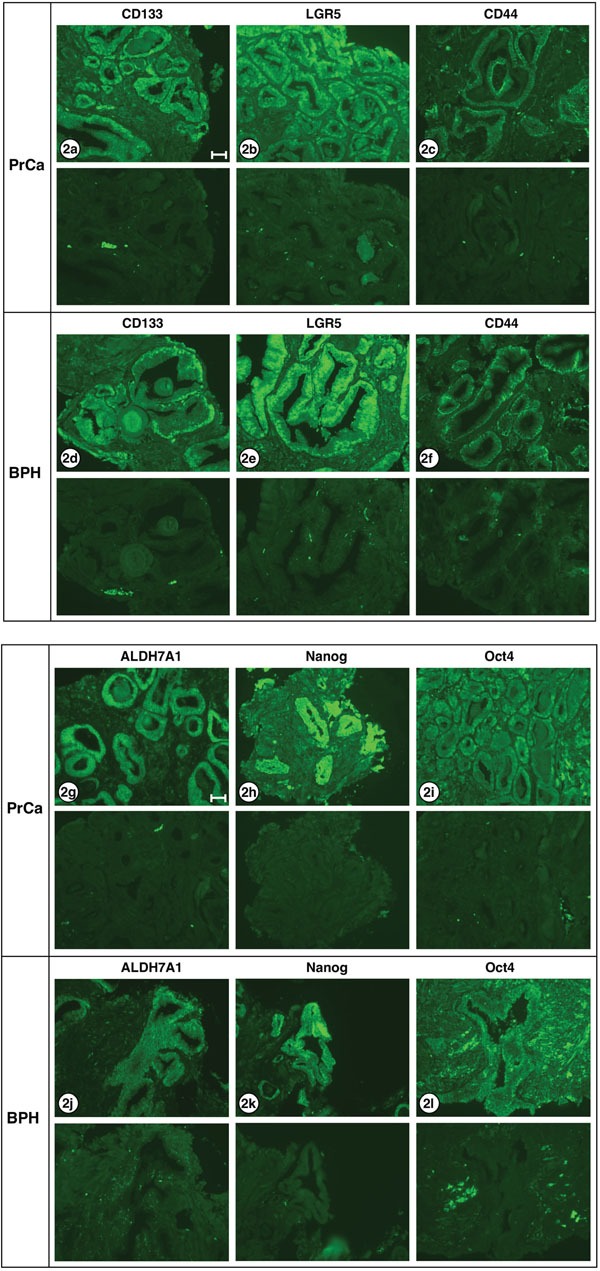
Stem cell markers expressed in PrCa and BPH Six different antibodies known to react with stem cells were examined for staining carcinoma and hyperplastic tissues: **a, d.** CD133; **b, e.** LGR5; **c, f.** CD44; **g, j.** ALDH7A1; **h, k.** Nanog; **i, l.** Oct4. The lower half of each frame shows the corresponding negative stain. The only differences in staining pattern were: CD133 appears to stain both cytoplasm and nucleus of carcinoma cells, but only the nucleus of hyperplastic cells. Oct4 stains both cytoplasm and nucleus of carcinoma cells, but only the cytoplasm of hyperplastic cells. ALDH7A1 shows weak nuclear staining and strong cytoplasmic staining in adenocarcinoma cells, but shows no detectable nuclear staining in hyperplastic cells. All exposures were 3 sec except ALDH7A1 and Nanog which were 1 sec. Images shown are from the following patient biopsie sections: 2a, A1; 2b, K1; 2c, C1; 2d, A1; 2e, K1; 2f, E1; 2g, C1; 2h, F1; 2i, C1; 2j, K1; 2k, C1; 2l; J1. Bar = 50 μm.

Figure 2 shows serial sections of needle biopsy tissue displaying carcinoma or hyperplasia, or both, reacted with antibodies specific for the stem cell markers CD133, LGR5, CD44, ALDH7A1, NANOG and Oct4. Sections derived from three patient tissue blocks are shown, patients A1, C1 and E1; 7 of 8 patient biopsies used in this study have yielded similar results. One patient sample – I1 – exhibited only hyperplasia. All six stem cell-specific antibodies stained the carcinoma or hyperplasia structures (top pictures in each pair) while non-immune antibody isotypes, used as negative controls (lower frames), showed no reactivity. Differences in the staining pattern of three antibodies were observed.

**Figure 3:**
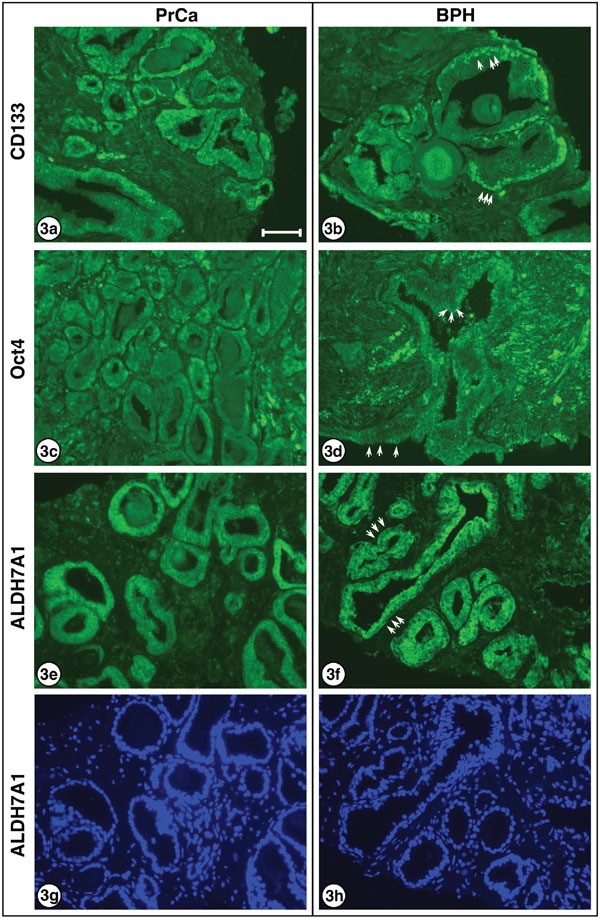
Higher resolution for CD133, Oct4 and and ALDH7A1 in PrCa and BPH Higher power images are presented for: CD133 **a.** carcinoma and **b.** hyperplasia, showing decreased cytoplasmic localization in BPH sections. This is readily noted by the prominent nuclei visible in (b), several of which are indicated by white arrows, which cannot be clearly discerned in (a) where cells exhibit relatively uniform cytoplasmic/nuclear staining. Oct4 **c.** carcinoma and **d.** hyperplasia, showing decreased nuclear localization in BPH sections. This is most easily observed by the appearance of clearly visible non-staining nuclei in (d), with examples shown by white arrows, surrounded by the brightly stained cytoplasm. ALDH7A1 **e.** carcinoma and **f.** hyperplasia, showing decreased nuclear localization in BPH sections. Again, this is most easily observed by the appearance of visible non-staining nuclei in (f), with examples indicated by white arrows, surrounded by brightly stained cytoplasm. Although the inverse nuclei are visible in (e), they are much more prominent in (f). DAPI-stained ALDH7A1 **g.** carcinoma and **h.** hyperplasia, showing identical DAPI-stained images as for (e) and (f) above. Note that nuclei of cells clearly staining in (e) and (f) are visible, as well as additional nuclei of cells in the sections that do not stain positively for ALDH7A1. 3a and 3b are from patient sample A1. 3c and 3e are from patient sample C1. 3d is from patient sample J1, and 3f is from patient sample E1. Bar = 50 μm.

For three of the proteins examined – LRG5, CD44 and Nanog – no obvious differences were noted in the nuclear/cytoplasmic staining of PrCa versus BPH sections. However, higher power views of the staining of carcinomatous and hyperplastic regions with the three antibodies to CD133, Oct4 and ALDH7A1 revealed changes in PrCa versus BPH sections which may be significant. As shown in Figure 3, CD133 expression is evident in both the nuclei and the cytoplasm of carcinoma cells but is largely absent from the cytoplasm of the hyperplastic cells (3a, carcinoma; 3b, hyperplasia). The Oct4 transcription factor is localized to both the nucleus and cytoplasm of carcinoma cells, but is largely absent from the nuclei of hyperplastic cells (3c, carcinoma; 3d, hyperplasia). ALDH7A1 also exhibited differential localization; while cytoplasmic staining was observed for both PrCa and BPH sections, the nuclear staining was greater in the PrCa sections and decreased in the BPH section (3e, carcinoma; 3f, hyperplasia). These differences are further considered in the Discussion.

Expression of all six stem cell-associated markers in the biopsied carcinoma cells provides evidence of their stemness as CSCs. The location of the expression of the markers CD133, in the plasma membrane and the endoplasmic reticulum in stem cells, [17] and Oct4, expressed in the nucleus [18] suggests that while adenocarcinoma cells consist of pluripotent CSCs, hyperplasia consists of differentiating cells. This conclusion agrees with the failure to establish proliferating cell cultures from human BPH whereas proliferating cultures can be readily established from carcinomas [7].

## DISCUSSION

### Detection of six stem cell-specific antigens in PrCa sections

We have shown the rather surprising result that many of the cells of the glands of “well-differentiated” adenocarcinoma of the prostate express six antigens, CD133, LGR5, CD44, ALDH7A1, NANOG and Oct4, antigens that have been shown to be “stem cell” markers in a variety of systems as discussed further below. The tissues we analyzed were residual from routine needle core biopsies of the prostate that had been processed into paraffin blocks by standard surgical pathology protocols. All yielded a diagnosis of adenocarcinoma or hyperplasia; however, samples were anonymized, so no further prognostic information or clinical follow-up was obtainable.

These new results build on our earlier work in which surgical specimens from prostatectomy cases, including adenocarcinomas, were cultured using hormone-free media and shown to proliferate as spheres/spheroids in suspension cultures [7]. These cultured cells also reproduced human prostate cancers when transplanted orthotopically into the anterior prostate lobes of SCID mice. Significantly, these cultured human prostate adenocarcinomas also contained cells with stem-like properties as shown by expression of CD133, CD44, ALDH7A1, CK5/14 and α2β1 antigens.

### Localization of CD133, Oct4 and ALDH7A1 and expression of LGR5, CD44 and NANOG

In the present work, we have examined CD133, CD44 and ALDH7A1 which were included in the prior study of propagated PrCa spheroids, as well as LGR5, NANOG and Oct4, which were not tested in this earlier work [7]. CD133, a pentaspan transmembrane glycoprotein also known as prominin 1, was chosen for examination as it is frequently used as a marker of CSCs [19–22]. Most recently, in an examination of glioblastoma-initiating cells, CD133 expression was shown to correlate with neural stem cells and pluripotent stem cells, possibly enabling differentiation into both neural and mesodermal cell types [23] However, in one flow cytometric analysis of PrCa stem-like cells, tumorigenicity correlated to a greater extent with side population (SP) cells rather than CD133 expression [24].

Oct4 is a nuclear POU homeodomain transcription factor, also known as Pou Class 5 homeobox 1 (POU5F1), and represents another classic protein marker for stemness and pluripotency [18, 25, 26]. As noted above, Oct4 localized to both the nucleus and cytoplasm of carcinoma cells, but was largely absent from the nuclei of hyperplastic cells (Figure 3c, 3d). A recent study of cervical cancer stem-like cells concluded that nuclear Oct4A serves as the driving force of cancer metastasis and recurrence, while cytoplasmic Oct4B may cooperate to regulate cancer progression [27]. The antiserum (Santa Cruz Biotech sc-9081) used in our expression study here detects Oct4A and is non cross-reactive with Oct4B.

The role of aldehyde dehydrogenases, for which there are some 19 human genes, in human pathogenesis has been much studied both in terms of cancer overall but specifically with regard to prostate cancer and cancer stem cells [28–30]. The isoform ALDH7A1 which we examined has been described as a marker of the CSC-like signature of prostate cancer cells, and as functionally involved in prostate cancer bone metastasis [31]. Although we observed ALDH7A1 to be more prominently expressed in the nuclei of PrCa cells in comparison with BPH (Figure 3e, 3f), we are unsure of the significance of this observation nor have we seen a similar report elsewhere.

The demonstration of LGR5, CD44 and NANOG in the PrCa sections examined here is also significant. LGR5, or Leucine-rich repeat containing G protein-coupled Receptor 5, is a member of the G protein-coupled 7-transmembrane GPRC family which figures prominently in the control of Wnt signaling pathways and in the maintenance of adult intestinal stem cells [32–34]. Significantly, LGR5 is expressed in a rare population of prostatic epithelial progenitor cells, and serves as a marker of prostatic stem cells. CD44 is a cell-surface glycoprotein and receptor for hyaluronic acid that is involved in hematopoiesis and tumorigenesis, and associated with stemness-associated properties of CSCs including self-renewal [35, 36]. Recently, CD44 was shown to play a key role to initiate EMT involved in luminal PrCa cell metastasis [37]. Lastly, the homeobox transcription factor NANOG represents a well studied protein involved in the self-renewal and pluripotency of stem cells [25, 38, 39]. Expression of NANOG, particularly in conjunction with HIF-1α, was proposed as a biomarker for prostate cancer [40].

As noted previously, three of the six markers examined – LRG5, CD44 and Nanog – exhibited similar staining in PrCa versus BPH sections. However, the staining with antibodies to CD133, Oct4 and ALDH7A1 revealed differences in PrCa versus BPH sections which appear to be significant in their nuclear/cytoplasmic expression. Further research will be required to characterize and understand how these differences allow propagation of truly cancerous cells versus cells that are merely hyperplastic.

### Origin of prostate cancer and the plasticity of stem cells

The development of PrCSCs in cancer tissue has been an enigma [7]. One possible source of the PrCSCs involves re-expression of embryonic genes by cancer cells via a re-programming process [41]. Alternatively, the cells that are subject to the carcinogenic process may arise from stem cells of the basal layer of the prostate gland. Pathology residents in training are traditionally taught that normal prostate glands have two layers: basal cells and epithelial cells, whereas cancer glands only have one layer: “epithelial” cells. Yet, this may represent a conceptual oversimplification. The undeniable appearance of a single cell layer in the glands of adenocarcinoma may result from mutation(s) that cause increased expansion of cells originating from the basal cell layer without a comparable expansion of normal epithelial cells. Thus, what is traditionally viewed as malignant epithelial cells may in fact represent malignant basal cells expressing stem cell properties and containing a subpopulation of true PrCSCs.

The association of prostate cancer induction with CSC proliferation has been suggested in many published reports. Also, since CSCs in many cancers may continue to proliferate and differentiate *in situ*, *e.g.* in breast, brain, prostate, gut, lung and head and neck cancers, a theory of a “Moving Target” [42] may be more aptly described as a “Vanishing Cancer Stem Cell.” Many current therapies treat the bulk of the differentiated, proliferating tumor mass without eliminating the initiating cells of origin, leading to frequent recurrence [43]. This is particularly relevant if one considers the plasticity of stem cells and poses the metaphysical question: what is a stem cell? The hematopoietic stem cell of the bone marrow, one well-known example, undergoes self-renewal as well as asymmetric cell division to produce the precursors of red cells, platelets, lymphocytes, monocytes and so forth. Most of the cell division occurs, not in the stem cell population, but in cells on one of these differentiation pathways known as transit amplifying cells. At each stage of maturation, previously multipotent cells restrict their differentiation potential until only one of these end cells is produced [44, 45]. Tissue renewal in other tissues does not always follow this well-ordered paradigm. For example, in the intestinal epithelium, two sources of stem cells have been described in small intestinal crypts: cycling LGR5-positive crypt base columnar cells and quiescent cells in the +4 position [46–48].

In the adult prostate, the epithelial stem cell is thought to reside in the basal layer of normal glands [49, 50], giving rise to epithelial cells that secrete, among other things, PSA, a serine protease important for dissolving coagulated semen. The “epithelial” cells of “well-differentiated” adenocarcinoma express a number of different properties: they may divide more rapidly than normal epithelial cells; they are capable of migration, a “mesenchymal” property; they can form glands out in the stroma without being anchored to a basement membrane; they can invade other normal tissues, both locally and metastatically. To do this they must activate expression of several groups of genes not normally expressed by epithelial cells, for example to dissolve basement membranes or to undergo extravasation. The term “well-differentiated adenocarcinoma” cell therefore does not imply that the cells are genetically or biochemically uniform, only that they do not look bizarre histologically. They are, in fact, “maldifferentiated.” The expression of the stem-cell markers that we have demonstrated may therefore reflect the process of genetic reregulation that these cells are undergoing. They may all be derived by mutation of preexisting, androgen independent, epithelial stem-cells in the basal layer that would normally express some of these antigens at some point in their cell cycle (more like the bone marrow model.) Or, they may be undergoing a process of reregulation to acquire stem-like properties (more like the intestinal epithelium model.) Both of these possibilities would be dependent on critical mutations to generate the cancer phenotype. In the case of normal tissue renewal, different tissues exhibit different strategies in which differentiated cells may reacquire “stem cell” properties. Expression of the antigens we have demonstrated in prostate adenocarcinoma cells has not been systematically studied in all these different tissue systems. To decide whether or not to call a cell with certain capacities for division, differentiation, and antigen expression a “stem-like” cell is therefore complex.

In our prior experiments, the frequency of PrCSCs *that would grow out in culture* was low, about 10^−7^, while in the current experiments, the frequency of prostate adenocarcinoma cells *in vivo* that express these “stem-cell markers,” appears in some fields (Figure 2, 3) to be quite high. Why might this be so? First, the ability of cells to grow in tissue culture and the expression of antigens *in vivo* test two different properties. As noted above, the typical adult gland consists of two layers of cells: a basal layer and an epithelial layer. In “well-differentiated” adenocarcinoma, the glands appear to be composed of only one layer of “epithelial” cells. Typical, normal basal cells and basement membranes are absent because the malignant “epithelial” cells invade the stroma and do not need to adhere to basement membranes to avoid apoptosis, a critical step in the development of malignancy. One should not conclude that all the histologically similar “epithelial” cells of “well-differentiated” adenocarcinoma have exactly the same properties. We would propose that a subset represent true prostatic epithelial CSCs, while others may express some but not all properties of PrCSCs.

Lastly, we note that while only a small percentage of cancer cells will grow out in tissue culture and display stem-like properties [7], the antibody markers suggest that a considerable rearrangement of metabolic pathways is associated with the development of both hyperplasia and cancer. Two different measurements of stem-like properties give different results. This indicates that our understanding of stemness and cellular plasticity is still in its infancy.

## CONCLUSIONS

Despite the heuristic appeal of the results presented here and their overall agreement with our previous studies [7], nonetheless, we wish to note several limitations which will only be overcome by further experimental research. First, as these results were obtained with anonymized formalin-fixed and paraffin-embedded sections, prepared solely for pathological analysis, there is no way to propagate living cells from these samples. Second, due to the limited number of sections that can be obtained from these samples, only a relatively small number of markers can be examined, an analysis which must necessarily be viewed as more qualitative than quantitative. Notwithstanding, we can conclude unambiguously that significant numbers of cells within clinical samples that serve as the pathological basis for a diagnosis of PrCa exhibit positive staining for six prominent markers of stem cells. We further conclude that all of these cells manifest at least some notable characteristics and properties of CSCs, and that within these populations reside *bona fide* PrCSCs that serve as the basis of human disease.

## MATERIALS AND METHODS

Sections of 5 μm thickness were cut from paraffin-embedded blocks containing patient needle biopsy samples. Sections were deparaffinized sequentially with xylene, 100% ethanol, 95% ethanol and 70% ethanol. After washing the sections with DI H_2_O, antigen retrieval is performed by boiling the sections in a mixture of 0.1M sodium citrate, 0.1M citric acid, and DI H_2_O in a microwave with high-heat setting. When the sections cooled down, they were washed with DI H_2_O followed by PBS. Sections were permeabilized with 0.2% Tween 20 in 5% donkey serum, and washed with PBS. Afterwards they were blocked with 5% donkey serum in PBS, and washed with PBS. Sections were then incubated with primary antibodies overnight at 4°C. Negative controls were also included by using isotype controls.

The primary antibodies used include rabbit anti-CD133 (Santa Cruz Biotech sc-30220) at 8 μg/ml, mouse anti-CD44 (BD Pharmingen 550392) at 10 μg/ml, rabbit anti-ALDH7A1 (Abgent AJ1002A) at 4 μg/ml, rabbit anti-LGR5 (Santa Cruz Biotech sc-135238) at 8 μg/ml, goat anti-Nanog (R&D Systems AF1997) at 8 μg/ml, and rabbit anti-Oct4 (Santa Cruz Biotech sc-9081) at 4 μg/ml. Sections were washed with PBS, and then incubated with appropriate secondary antibodies at room temperature for an hour in dark environment. The secondary antibodies used include Alexa Fluor 488 goat anti-rabbit (Jackson ImmunoResearch 111-545-003) at 15 μg/ml, Alexa Fluor 488 donkey anti-goat (Jackson ImmunoResearch 705-545-003) at 15 μg/ml, and Alexa Fluor 488 donkey anti-mouse (Jackson ImmunoResearch 715-545-15) at 7.5 μg/ml.

After washing with PBS in the dark, counterstain ProLong Gold Antifade Reagent with DAPI (Invitrogen P36935) was applied onto the sections. Sections were left to dry overnight and then observed under a fluorescence microscope, Nikon Eclipse E800. Pictures of stained sections were taken under 20x objective with a typical exposure time of 3 sec.

## References

[1] Hanahan D, Weinberg RA (2011). Hallmarks of cancer: the next generation. Cell.

[2] Wulf G, Garg P, Liou YC, Iglehart D, Lu KP (2004). Modeling breast cancer *in vivo* and ex vivo reveals an essential role of Pin1 in tumorigenesis. EMBO J.

[3] Lawson DA, Zong Y, Memarzadeh S, Xin L, Huang J, Witte ON (2010). Basal epithelial stem cells are efficient targets for prostate cancer initiation. Proc Natl Acad Sci U S A.

[4] Feldman BJ, Feldman D (2001). The development of androgen-independent prostate cancer. Nat Rev Cancer.

[5] Mundy GR (2002). Metastasis to bone: causes, consequences and therapeutic opportunities. Nat Rev Cancer.

[6] Signoretti S, Loda M (2006). Defining cell lineages in the prostate epithelium. Cell Cycle.

[7] Finones RR, Yeargin J, Lee M, Kaur AP, Cheng C, Sun P, Wu C, Nguyen C, Wang-Rodriguez J, Meyer AN, Baird SM, Donoghue DJ, Haas M (2013). Early human prostate adenocarcinomas harbor androgen-independent cancer cells. PLoS One.

[8] Lang SH, Sharrard RM, Stark M, Villette JM, Maitland NJ (2001). Prostate epithelial cell lines form spheroids with evidence of glandular differentiation in three-dimensional Matrigel cultures. Br J Cancer.

[9] Smith BA, Sokolov A, Uzunangelov V, Baertsch R, Newton Y, Graim K, Mathis C, Cheng D, Stuart JM, Witte ON (2015). A basal stem cell signature identifies aggressive prostate cancer phenotypes. Proc Natl Acad Sci U S A.

[10] Takahashi K, Tanabe K, Ohnuki M, Narita M, Ichisaka T, Tomoda K, Yamanaka S (2007). Induction of pluripotent stem cells from adult human fibroblasts by defined factors. Cell.

[11] Kawasaki H, Ogura H, Arai Y, Baba S, Kosugi I, Tsutsui Y, Iwashita T (2010). Aggressive progression of breast cancer with microscopic pulmonary emboli possessing a stem cell-like phenotype independent of its origin. Pathol Int.

[12] You H, Ding W, Rountree CB (2010). Epigenetic regulation of cancer stem cell marker CD133 by transforming growth factor-beta. Hepatology (Baltimore, Md).

[13] Le Magnen C, Bubendorf L, Rentsch CA, Mengus C, Gsponer J, Zellweger T, Rieken M, Thalmann GN, Cecchini MG, Germann M, Bachmann A, Wyler S, Heberer M, Spagnoli GC (2013). Characterization and clinical relevance of ALDHbright populations in prostate cancer. Clin Cancer Res.

[14] Becker L, Huang Q, Mashimo H (2010). Lgr5, an intestinal stem cell marker, is abnormally expressed in Barrett's esophagus and esophageal adenocarcinoma. Dis Esophagus.

[15] Kim S, Lim B, Kim J (2010). EWS-Oct-4B, an alternative EWS-Oct-4 fusion gene, is a potent oncogene linked to human epithelial tumours. Br J Cancer.

[16] Lu TY, Lu RM, Liao MY, Yu J, Chung CH, Kao CF, Wu HC (2010). Epithelial cell adhesion molecule regulation is associated with the maintenance of the undifferentiated phenotype of human embryonic stem cells. J Biol Chem.

[17] Curley MD, Therrien VA, Cummings CL, Sergent PA, Koulouris CR, Friel AM, Roberts DJ, Seiden MV, Scadden DT, Rueda BR, Foster R (2009). CD133 expression defines a tumor initiating cell population in primary human ovarian cancer. Stem Cells.

[18] Rizzino A, Wuebben EL (2016). Sox2/Oct4: A delicately balanced partnership in pluripotent stem cells and embryogenesis. Biochim Biophys Acta.

[19] Grosse-Gehling P, Fargeas CA, Dittfeld C, Garbe Y, Alison MR, Corbeil D, Kunz-Schughart LA (2013). CD133 as a biomarker for putative cancer stem cells in solid tumours: limitations, problems and challenges. J Pathol.

[20] Li Z (2013). CD133: a stem cell biomarker and beyond. Exp Hematol Oncol.

[21] Mak AB, Schnegg C, Lai CY, Ghosh S, Yang MH, Moffat J, Hsu MY (2014). CD133-targeted niche-dependent therapy in cancer: a multipronged approach. Am J Pathol.

[22] Li X, Zhao H, Gu J, Zheng L (2015). Prognostic value of cancer stem cell marker CD133 expression in pancreatic ductal adenocarcinoma (PDAC): a systematic review and meta-analysis. Int J Clin Exp Pathol.

[23] Pavon LF, Sibov TT, de Oliveira DM, Marti LC, Cabral FR, de Souza JG, Boufleur P, Malheiros SM, de Paiva Neto MA, da Cruz EF, Chudzinski-Tavassi AM, Cavalheiro S (2016). Mesenchymal stem cell-like properties of CD133+ glioblastomainitiating cells. Oncotarget.

[24] Zhou J, Wang H, Cannon V, Wolcott KM, Song H, Yates C (2011). Side population rather than CD133(+) cells distinguishes enriched tumorigenicity in hTERT-immortalized primary prostate cancer cells. Molecular cancer.

[25] Kashyap V, Rezende NC, Scotland KB, Shaffer SM, Persson JL, Gudas LJ, Mongan NP (2009). Regulation of stem cell pluripotency and differentiation involves a mutual regulatory circuit of the NANOG, OCT4, and SOX2 pluripotency transcription factors with polycomb repressive complexes and stem cell microRNAs. Stem Cells Dev.

[26] Pan GJ, Chang ZY, Scholer HR, Pei D (2002). Stem cell pluripotency and transcription factor Oct4. Cell Res.

[27] Li SW, Wu XL, Dong CL, Xie XY, Wu JF, Zhang X (2015). The differential expression of OCT4 isoforms in cervical carcinoma. PLoS One.

[28] Kozovska Z, Gabrisova V, Kucerova L (2016). Malignant melanoma: diagnosis, treatment and cancer stem cells. Neoplasma.

[29] La Porta CA (2012). Thoughts about cancer stem cells in solid tumors. World J Stem Cells.

[30] Tirino V, Desiderio V, Paino F, De Rosa A, Papaccio F, La Noce M, Laino L, De Francesco F, Papaccio G (2013). Cancer stem cells in solid tumors: an overview and new approaches for their isolation and characterization. FASEB J.

[31] van den Hoogen C, van der Horst G, Cheung H, Buijs JT, Pelger RC, van der Pluijm G (2011). The aldehyde dehydrogenase enzyme 7A1 is functionally involved in prostate cancer bone metastasis. Clin Exp Metastasis.

[32] Barker N, Tan S, Clevers H (2013). Lgr proteins in epithelial stem cell biology. Development.

[33] de Lau W, Peng WC, Gros P, Clevers H (2014). The R-spondin/Lgr5/Rnf43 module: regulator of Wnt signal strength. Genes Dev.

[34] Koo BK, Clevers H (2014). Stem cells marked by the R-spondin receptor LGR5. Gastroenterology.

[35] Chanmee T, Ontong P, Kimata K, Itano N (2015). Key Roles of Hyaluronan and Its CD44 Receptor in the Stemness and Survival of Cancer Stem Cells. Front Oncol.

[36] Horta S, Agostinho AL, Mateus R, Pereira L, Pereira C, Capinha L, Doktorovova S, Brito A, Videira M (2015). Looking out for cancer stem cells' properties: the value-driving role of CD44 for personalized medicines. Curr Cancer Drug Targets.

[37] Shang Z, Cai Q, Zhang M, Zhu S, Ma Y, Sun L, Jiang N, Tian J, Niu X, Chen J, Sun Y, Niu Y (2015). A switch from CD44(+) cell to EMT cell drives the metastasis of prostate cancer. Oncotarget.

[38] Gawlik-Rzemieniewska N, Bednarek I (2016). The role of NANOG transcriptional factor in the development of malignant phenotype of cancer cells. Cancer Biol Ther.

[39] Jeter CR, Yang T, Wang J, Chao HP, Tang DG (2015). Concise Review: NANOG in Cancer Stem Cells and Tumor Development: An Update and Outstanding Questions. Stem Cells.

[40] Miyazawa K, Tanaka T, Nakai D, Morita N, Suzuki K (2014). Immunohistochemical expression of four different stem cell markers in prostate cancer: High expression of NANOG in conjunction with hypoxia-inducible factor-1alpha expression is involved in prostate epithelial malignancy. Oncol Lett.

[41] Monk M, Holding C (2001). Human embryonic genes re-expressed in cancer cells. Oncogene.

[42] Nakano I (2015). Stem cell signature in glioblastoma: therapeutic development for a moving target. J Neurosurg.

[43] Seiwert TY, Fayette J, Cupissol D, Del Campo JM, Clement PM, Hitt R, Degardin M, Zhang W, Blackman A, Ehrnrooth E, Cohen EE (2014). A randomized, phase II study of afatinib versus cetuximab in metastatic or recurrent squamous cell carcinoma of the head and neck. Ann Oncol.

[44] Attema JL, Papathanasiou P, Forsberg EC, Xu J, Smale ST, Weissman IL (2007). Epigenetic characterization of hematopoietic stem cell differentiation using miniChIP and bisulfite sequencing analysis. Proc Natl Acad Sci U S A.

[45] Mangel M, Bonsall MB (2008). Phenotypic evolutionary models in stem cell biology: replacement, quiescence, and variability. PLoS One.

[46] Barker N, van Es JH, Kuipers J, Kujala P, van den Born M, Cozijnsen M, Haegebarth A, Korving J, Begthel H, Peters PJ, Clevers H (2007). Identification of stem cells in small intestine and colon by marker gene Lgr5. Nature.

[47] Barker N, van Oudenaarden A, Clevers H (2012). Identifying the stem cell of the intestinal crypt: strategies and pitfalls. Cell Stem Cell.

[48] Zhang N, Yantiss RK, Nam HS, Chin Y, Zhou XK, Scherl EJ, Bosworth BP, Subbaramaiah K, Dannenberg AJ, Benezra R (2014). ID1 is a functional marker for intestinal stem and progenitor cells required for normal response to injury. Stem Cell Reports.

[49] Lam JS, Reiter RE (2006). Stem cells in prostate and prostate cancer development. Urologic oncology.

[50] Zenzmaier C, Untergasser G, Berger P (2008). Aging of the prostate epithelial stem/progenitor cell. Exp Gerontol.

